# Supplementary material for: Towards unified AI-driven fracture mechanics: the extended deep energy method (XDEM)
Source: Nat Commun. 2026 Aug 15;17:8492. doi: 10.1038/s41467-026-76748-1 (PMC13484471; doi:10.1038/s41467-026-76748-1)
Supplement: Supplementary file 3 [file 41467_2026_76748_MOESM3_ESM.zip › pic/Results/Stress_intensity_factor/Mixed/beta_15/crack_detailed_fields.pdf]

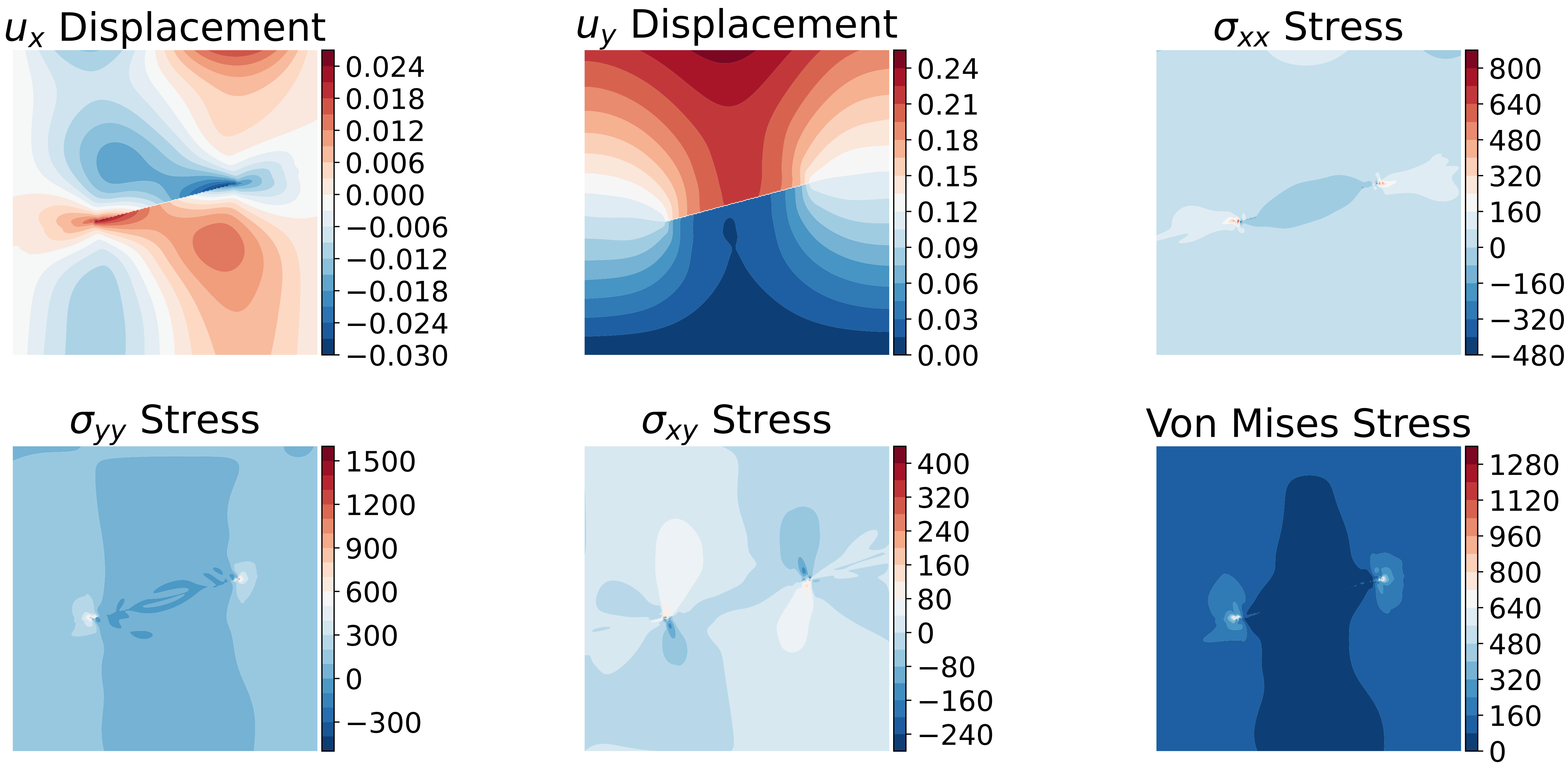
u_x Displacement
0.024
0.018
0.012
0.006
0.000
−0.006
−0.012
−0.018
−0.024
−0.030
u_y Displacement
0.24
0.21
0.18
0.15
0.12
0.09
0.06
0.03
0.00
σ_{xx} Stress
800
640
480
320
160
0
−160
−320
−480
σ_{yy} Stress
1500
1200
900
600
300
0
−300
σ_{xy} Stress
400
320
240
160
80
0
−80
−160
−240
Von Mises Stress
1280
1120
960
800
640
480
320
160
0
